# Airway Microbiome and Serum Metabolomics Analysis Identify Differential Candidate Biomarkers in Allergic Rhinitis

**DOI:** 10.3389/fimmu.2021.771136

**Published:** 2022-01-05

**Authors:** Yuze Yuan, Chao Wang, Guoqiang Wang, Xiaoping Guo, Shengyu Jiang, Xu Zuo, Xinlei Wang, Alan Chen-Yu Hsu, Mingran Qi, Fang Wang

**Affiliations:** ^1^ Department of Pathogeny Biology, College of Basic Medical Sciences, Jilin University, Changchun, China; ^2^ Priority Research Centre for Healthy Lungs, Hunter Medical Research Institute (HMRI), University of Newcastle, New Lambton Heights, NSW, Australia; ^3^ School of Medicine and Public Health, College of Health, Medicine and Wellbeing, University of Newcastle, Callaghan, NSW, Australia; ^4^ Programme in Emerging Infectious Diseases, Duke - National University of Singapore (NUS) Medical School, Singapore, Singapore

**Keywords:** allergic rhinitis, microbiome, metabolomics, biomarkers, multiomics

## Abstract

Allergic rhinitis (AR) is a common heterogeneous chronic disease with a high prevalence and a complex pathogenesis influenced by numerous factors, involving a combination of genetic and environmental factors. To gain insight into the pathogenesis of AR and to identity diagnostic biomarkers, we combined systems biology approach to analyze microbiome and serum composition. We collected inferior turbinate swabs and serum samples to study the microbiome and serum metabolome of 28 patients with allergic rhinitis and 15 healthy individuals. We sequenced the V3 and V4 regions of the 16S rDNA gene from the upper respiratory samples. Metabolomics was used to examine serum samples. Finally, we combined differential microbiota and differential metabolites to find potential biomarkers. We found no significant differences in diversity between the disease and control groups, but changes in the structure of the microbiota. Compared to the HC group, the AR group showed a significantly higher abundance of 1 phylum (*Actinobacteria*) and 7 genera (*Klebsiella*, *Prevotella* and *Staphylococcus*, *etc.*) and a significantly lower abundance of 1 genus (*Pelomonas*). Serum metabolomics revealed 26 different metabolites (Prostaglandin D2, 20-Hydroxy-leukotriene B4 and Linoleic acid, *etc.*) and 16 disrupted metabolic pathways (Linoleic acid metabolism, Arachidonic acid metabolism and Tryptophan metabolism, *etc.*). The combined respiratory microbiome and serum metabolomics datasets showed a degree of correlation reflecting the influence of the microbiome on metabolic activity. Our results show that microbiome and metabolomics analyses provide important candidate biomarkers, and in particular, differential genera in the microbiome have also been validated by random forest prediction models. Differential microbes and differential metabolites have the potential to be used as biomarkers for the diagnosis of allergic rhinitis.

## 1 Introduction

Allergic rhinitis (AR) is a common heterogeneous chronic disorder in both children and adults that currently affects up to 40% of the global population, with a high prevalence especially in industrialized countries, and has a negative impact on the social life, school performance and productivity of patients (1, 2). It is an inflammation disease of the nasal mucosa characterized by the presence of one or more nasal symptoms, including nasal pruritus, sneezing, rhinorrhea, and nasal congestion (3–5). Its pathogenesis is complex and influenced by numerous factors, involving a combination of genetic and environmental factors, in which the interaction between a dysregulated state of the microbiota and an allergic response to allergen exposure has a major role (6). There are no biomarkers available for clinical practice to predict the subtype and severity of AR and the development of its common comorbidities (5).

The respiratory tract is a complex system that extends from the nasal cavity, nasopharynx, oropharynx, and trachea straight to the lungs and is divided into the upper respiratory tract and the lower respiratory tract. It has been found that a large number of bacterial communities exist throughout the respiratory tract, and the microbial community structure has variations in richness and diversity along the respiratory tract (7), which has a great role in determining the occurrence and development of diseases (8–10). Current studies have found that lower respiratory microflora can be involved in the development of lung diseases such as lung cancer (11), asthma (12, 13) and chronic obstructive pulmonary disease (14), by modulating the local inflammatory response. The upper respiratory tract is composed of different anatomical structures that have different epithelial cell types and are exposed to various environmental factors, so the nasal microbiota may be abnormal, especially in the context of airway allergy (7). Therefore, exploring the relationship between allergic inflammation and the upper airway microbiota is essential to understand the mechanisms associated with AR and to provide potential therapeutic strategies.

Metabolomics is the detection, identification and quantification of small molecule compounds involved in metabolism, and its most widespread application is the identification of biomarkers for diagnosis and prediction of diseases, which have great potential in the elucidation of disease mechanisms (15–17). In some respiratory diseases, such as asthma (18, 19), lung cancer (20), and chronic obstructive pulmonary disease (21), metabolomics approaches have identified potential biomarkers that can be used as disease characterization and new therapeutic targets (22, 23). Our previous study also identified differences in metabolic profiles between asthma phenotypes (24). Environmental factors and metabolic markers in allergic rhinitis might be new points to explore metabolic changes in allergic diseases and to identify some potential metabolites and key metabolic pathways to account for the pathogenesis of AR (25).

In this study, 16S rRNA sequencing was used to identify the microorganisms of the nasal airways of AR patients and healthy volunteers to uncover the role of microflora in the pathogenesis of AR. We also conducted an in-depth exploration of the differential metabolic status of AR patients and healthy volunteers by non-targeted metabolomics to elucidate the metabolic characteristics and metabolic pathway patterns of AR patients and to further understand the pathogenesis of AR by multi-omics association analysis.

## 2 Materials and Methods

### 2.1 Study Population and Sample Collection

We recruited patients with acute exacerbations of allergic rhinitis at the China-Japan Union Hospital of Jilin University during the spring and fall of April 2018 to May 2019. Inferior turbinate mucosa samples from the patients and age- and sex- matching healthy subjects were obtained. Allergic rhinitis patients were diagnosed according to the following criteria: the occurrence of 2 or more symptoms of sneezing, clear watery nasal discharge, itchy nose and nasal congestion that persist or accumulate for more than 1h per day, accompanied by ocular symptoms such as itchy eyes, tearing and red eyes; nasal endoscopy of the nasal mucosa showing pallor and edema; seasonal allergen test result positive for serum-specific IgE (26). The exclusion criteria used for the above patients were as follows: receiving no immunotherapy; not diagnosed with malignancy; no history of allergic skin disease or allergic asthma; receiving no medication for allergic diseases in the 2 months prior to sampling. Healthy controls without clinical symptoms of rhinitis were enrolled and paired.

This study was performed in accordance with the Helsinki Declaration and Rules of Good Clinical Practice. It was approved by the China-Japan Union Hospital of Jilin University Ethics Committee (2018-NSFC-029) and registered in Chinese Clinical Trial Registry (NO. ChiCTR1800015420). All participants signed written informed consent.

### 2.2 DNA Extraction and 16S rDNA Sequencing

All inferior turbinate samples were collected and immediately frozen at -80°C. Total genome DNA from samples was extracted using CTAB method. 16S rRNA genes of distinct regions (16S V3-V4) were amplified used specific primer (341F—CCTAYGGGRBGCASCAG and 806R—GGACTACNNGGGTATCTAAT). After PCR products mixing and purification, sequencing libraries were generated using Ion Plus Fragment Library Kit 48 rxns (Thermo Scientific), whose quality was assessed on the Qubit@ 2.0 Fluorometer (Thermo Scientific), and finally library was sequenced on an Ion S5^TM^ XL platform.

### 2.3 Sample Preparation for Metabolomics and UPLC-Q/TOF-MS/MS Procedure

All serum samples were stored in aliquots at -80°C and thawed on ice prior to analysis. The serum (400 μL) of each sample was mixed with acetonitrile (1,200 μL) and vortexed for 3 min. After 15 min of resting on ice, all mixtures were centrifuged at 12,000 g for 10 min at 4°C in order to remove proteins. Next the supernatant was collected and lyophilized at -60°C and 10 pa air pressure for 20 h. The lyophilized material was redissolved in 150 μL 90% methanol and then centrifuged for the supernatant as test sample solution. The quality control (QC) sample was a 5 μL aliquot of each test sample solution that was mixed for method validation.

The UPLC-QTOF-MS analysis was performed by Waters Xevo G2-XS QTOF mass spectrometer (Waters Co., Milford, MA, USA.) combined with a UPLC system through an electrospray ionization (ESI) interface. ACQUITY UPLC BEH C18 (50 mm × 2.1 mm, 1.7 μm, Waters Co., Milford, MA, USA.) was used for chromatographic separation. The column temperature was set at 30°C and the room temperature was set at 16°C. The mobile phase consisted of eluent A (0.1% formic acid in aqueous solution) and eluent B (0.1% formic acid in acetonitrile solution). The gradient elution program was set up as follows: 0~2 min, 10% B; 2~26 min, 10%→90% B; 26~28 min, 90% B; 28~28.1 min, 90%→10% B; 28.1~30 min, 10% B with the flow rate at 0.4 mL/min. Mass spectra were obtained from 100 to 1200 Da in MSE centroid mode.

The optimized MS parameters were shown as follows based on our previous study (27, 28): cone voltage (40 V), capillary voltage at 2.6 kV (ESI+ mode) and 2.2 kV (ESI- mode), cone gas flow rate (50 L/h under the 120°C source temperature condition) and desolvation gas flow (800 L/h at 300°C desolvation temperature). In the MSE mode of mass spectrometry, low energy and high energy were set as 6 V and 20–40 V, respectively. Sodium formate solution was used to calibrate the instrument, and leucine enkephalin was used as the Lock Spray™ calibration standard liquid. The QC sample was randomly injected 6 times throughout the whole process. Data processing was performed on a MassLynx V4.1 workstation.

### 2.4 Microbiomics Study

All sequencing raw data were deposited into the NCBI Sequence Read Archive database (Accession number, PRJNA760816). Raw reads were processed into clean reads by data filtration using Cutadapt (v1.9.1) and chimera removal using UCHIME algorithm. The quality of all sequence have been filtered to ensure that the average quality of the bases was more than Q20. Sequences with more than 97% similarity were assigned to the same operational taxonomic units (OTUs). For clustered OTUs, the Silva Database based on Mothur algorithm was used to annotate taxonomic information. The alpha diversity and beta diversity analysis were calculated with QIIME software (version 1.9.1) and displayed with R software (version 2.15.3). Metastats analysis of phylum and genus was performed with R software, followed by false discovery rate correction. Random forest analysis was the classification of samples based on a machine learning model for the purpose of filtering variables.

### 2.5 Metabolomics Study

The raw data from the UPLC-Q/TOF-MS/MS system were processed with MarkerLynx (v4.1) software for alignment, deconvolution, and reduction to further multivariate analysis. The main parameters were set as follows: retention time range 0~28 min, mass range 100~1200 Da, retention time window 0.20 min, mass window 0.1 Da, marker intensity threshold 2000 counts and noise cancellation level 6. The processed data were entered into SIMCA-P software (Version 14.1) for multivariate analysis, including principal component analysis (PCA) and orthogonal projection to latent structures discriminant analysis (OPLS-DA). S-plots based on OPLS-DA predictions could demonstrate potential biomarkers which significantly contribute to metabolic differences. Metabolites with the variable importance in the projection (VIP) values above 1.0 and p-value below 0.05 were considered significantly different. The distinct metabolites were identified by comparing mass spectral fragmentation patterns according to HMDB databases(http://www.hmdb.ca/). According to the mobile phase, the adducts were selected as [M+H]+ and [M+Na]+ in ESI+, [M-H]- and [M+FA-H]-, with a mass tolerance of 10 ppm. Then, the predictive receiver operating characteristic (ROC) curve was used to estimate the accuracy of identified metabolites as potential biomarkers with the area under curve (AUC)>0.6. Potential biomarkers were screened through MetaboAnalyst 4.0 (http://www.metaboanalyst.ca/) for potential metabolic pathways, with the impact values above 0.10 and p values below 0.05.

### 2.6 Data Analysis

The results are presented as mean ± standard deviation (SD). Statistical analysis was performed using one-way analysis of variance (ANOVA) for multiple comparisons. Data normality was assessed by the Kolmogorov-Smirnov method. Comparisons between the two groups were performed by t-test. Student’s t-test was used to calculate p-value with homogeneity of variance; conversely, Welch’s t-test was used to calculate p-value. Mann-Whitney-Wilcoxon test was used to analyze non-normal data. All statistical analysis about the identification of distinct metabolites was completed with R (v4.0.2) basic statistical packages. All statistical significance was accepted at p<0.05.

## 3 Results

### 3.1 Characteristics of All Participants

A total of 43 participants with AR (n=28) and healthy controls (HC) (n=15) were enrolled in this study. Clinical demographics of the study cohort are presented in **Table 1**. The statistics of basic natural characteristics, including gender, age, and BMI values, did not display any significant difference between the two groups. The serum total IgE level was significantly higher in subjects with allergic rhinitis than in the healthy controls. All patients with allergic rhinitis are seasonal allergy patients with acute exacerbations.

**Table 1:** Clinical characteristics of the enrolled participants.

Parameter	AR	HC
No. of subjects	28	15
Male (%)	14 (50.0)	6 (40.0)
Female (%)	14 (50.0)	9 (60.0)
Serum IgE level (range: ≥200 IU/ml)	16 (57.1)	0 (0.0)
Serum IgE level (range: 100-199 IU/ml)	12 (42.9)	0 (0.0)
Serum IgE level (range: 0-99 IU/ml)	0 (0.0)	12 (100.0)
Age range, years	18-66	18-56
Mean age, years (SEM)	39.8(± 2.7)	34.8(± 3.2)
BMI, kg·m^-2^ (SEM)	23.2(± 0.6)	22.7 (± 0.7)
Seasonal Allergies	28	0

### 3.2 Microbiota in the Upper Respiratory Tract of AR Patients Versus HCs

#### 3.2.1 Estimation of Sequencing Depth

16S rDNA sequencing of 43 samples was based on the Ion S5^TM^ XL sequencing platform. After quality control filtering, an average of 79,137 valid data were obtained per sample. A total of 2,736 OTUs were obtained by clustering the sequences into OTUs with 97% identity, and then the sequences were annotated with Silva132. A total of 1,314 (48.03%) OTUs were annotated to the genus level in the annotation results.

**Figure 1:**
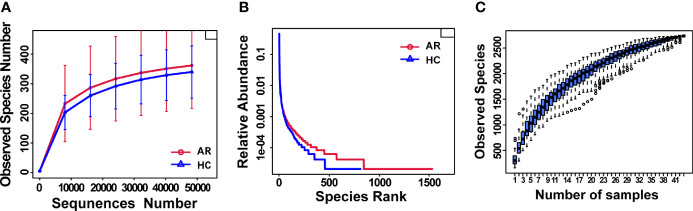
Estimation of sample depth in the AR and HC groups. **(A)** Rarefaction curves. **(B)** Rank Abundance curves. **(C)** Species Accumulation Boxplots. AR, allergic rhinitis; HC, healthy control.

Rarefaction curves showed curves in each group of samples reaching the platform stage, indicating a reasonable amount of sequencing data (**Figure 1A**). Rank Abundance curves reflected great richness and evenness in each set of samples (**Figure 1B**). The species accumulation boxplot showed a gradual increase in species diversity with increasing sample size, with the curves flattening out at 43 sample sizes (**Figure 1C**).

#### 3.2.2 Alpha-Diversity and Beta-Diversity

**Figure 2:**
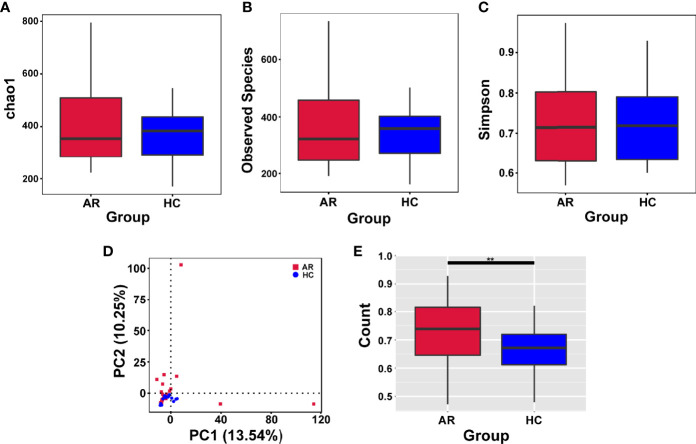
Alpha-diversity analysis of microbiome in allergic rhinitis (AR) group and healthy control (HC) group. **(A)** Chao1 index statistics. **(B)** Observed species index statistics. **(C)** Simpson index statistics. **(D)** Display of Principal Component Analysis (PCA) of the samples in two-dimension (PC1 = 13.54%, PC2 = 10.25%). **(E)** Beta diversity index statistics. Student’s t-test, **p < 0.01.

Alpha-diversity is used to analyze the diversity of microbial communities in a group. The assessment of the Chao1, Observed species and Simpson indexes, in each sample revealed that α-diversity was not changed in allergic rhinitis patients compared with the HCs (**Figures 2A–C**). In short, no significant change in species richness and diversity was observed.

Beta-diversity is a comparative analysis of the microbial community composition of different groups. Principal Component Analysis (PCA) based on Euclidean distances is able to extract the two axes that maximize the differences between samples, thus reflecting the differences in multidimensional data on a two-dimensional coordinate chart (**Figure 2D**). Beta diversity index analyzed by t-test showed significant differences between the two groups (**Figure 2E**).

#### 3.2.3 Distribution of Microbiota Taxonomic Composition in AR Patients

**Figure 3:**
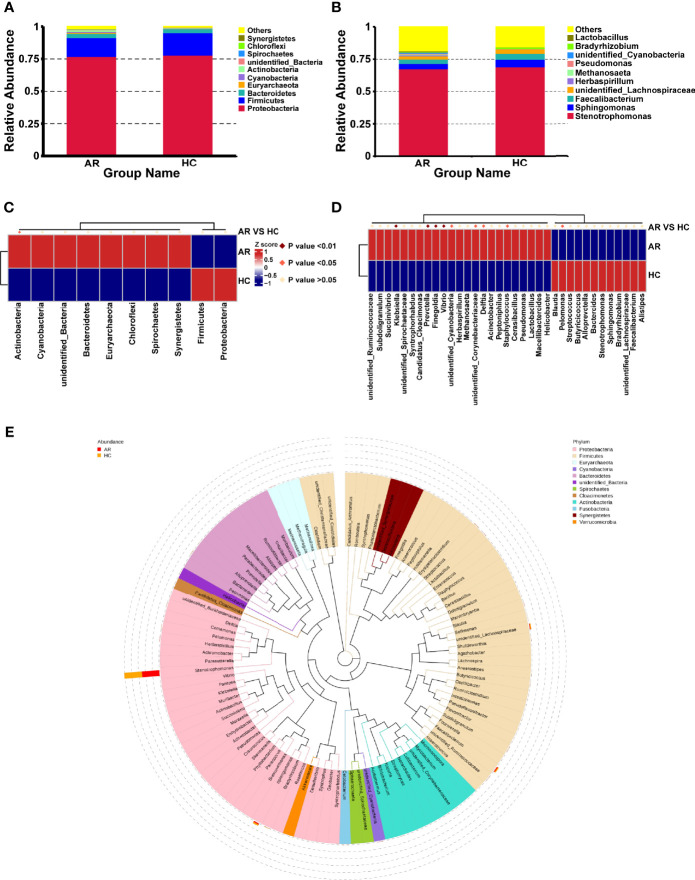
The distribution of taxa in phylum and genus levels of AR and HC groups. **(A)** composition of microbiome at the phylum level. **(B)** composition of microbiome at the genus level. **(C)** the statistical results of top 10 phyla. **(D)** the statistical results of top 35 genera. **(E)** Phylogenetic tree constructed from representative sequences of genus-level species, with branch and fan colors indicating their corresponding gates, and stacked histograms outside the fan rings indicating information on the abundance distribution of the genus in different samples.

According to the species annotation results, the top 10 species with the highest abundance at phylum and genus level are selected for AR and HC group to generate a relative abundance histogram, so as to visualize the species with higher relative abundance and their proportion at different taxonomic levels. At the phylum level, *Proteobacteria*, *Firmicutes*, and *Bacteroidetes* were the most abundant entities in the airway microbiota (**Figure 3A**). In addition, the *Stenotrophomonas*, *Sphingomonas*, and *Faecalibacterium* dominated the airway microbiota at the genus level (**Figure 3B**). There was higher abundance of the *Actinobacteria* phylum in ARs (p<0.05, **Figure 3C**). *Klebsiella*, *Prevotella*, *Finegoldia*, *Vibrio* were obviously significantly enriched in ARs (p<0.01, **Figure 3D**). And *unidentified_Cyanobacteria*, *unidentified_Corynebacteriaceae*, *Delftia*, *Staphylococcus* were higher in AR patients compared with HCs, while the *Pelomonas* was more abundant in HCs (p<0.05, **Figure 3D**). To further investigate the phylogenetic relationships of species at the genus level, a representative sequence of the genus top100 was obtained by multiple sequence alignment and is shown in **Figure 3E**.

#### 3.2.4 Predictive Modeling of the Airway Microbial Profile for AR

**Figure 4:**
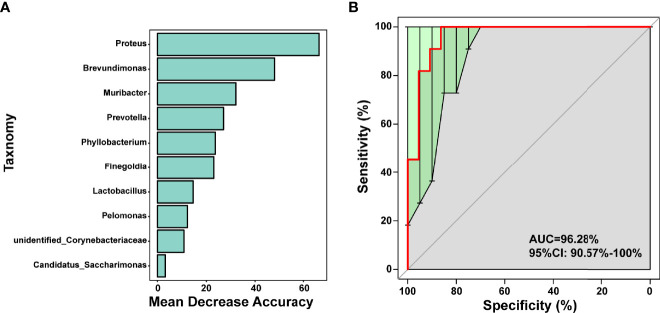
Prediction model of the airway microbiota for AR status based on the genus-level relative abundances using random forests. **(A)** variable importance ranking chart, MeanDecreaseAccuracy measures the degree to which the prediction accuracy of a random forest decreases by changing the value of a variable to a random number. A higher value indicates that the variable is more important. **(B)** ROC curve of the AR model using 10 discriminatory genera.

Random forest is a classical machine learning model based on classification tree algorithm that provided further support for the differentiation of AR and HC groups. This analysis based on OTU-based feature constructed random forest predictive model for the 10 genera. Significant genera were selected by MeanDecreaseAccuracy (**Figure 4A**), 10-fold cross-validation was done for the model and receiver operating characteristic (ROC) curves are plotted to score the predictive power. The area under the curve (AUC) was 0.9628 (95% CI: 0.906−1.000) (**Figure 4B**), suggesting that the airway microbiota had the potential to diagnose allergic rhinitis patients from healthy and disease controls. We observed that in the model, 10 genera were *Proteus*, *Brevundimonas*, *Muribacter*, *Prevotella*, *Phyllobacterium*, *Finegoldia*, *Lactobacillus*, *Pelomonas*, *unidentified_Corynebacteriaceae*, *Candidatus_Saccharimonas*, 5 belonged to the phylum *Proteobacteria*, 2 belonged to *Firmicutes*, 1 belonged to *Bacteroidetes*, and 1 belonged to *Actinobacteria*. Therefore, they might be used as bio-markers to identify patients most likely to develop AR.

### 3.3 Metabolomic Analysis of AR Versus HC

#### 3.3.1 Multivariate Analysis of Metabolomic Data

**Figure 5:**
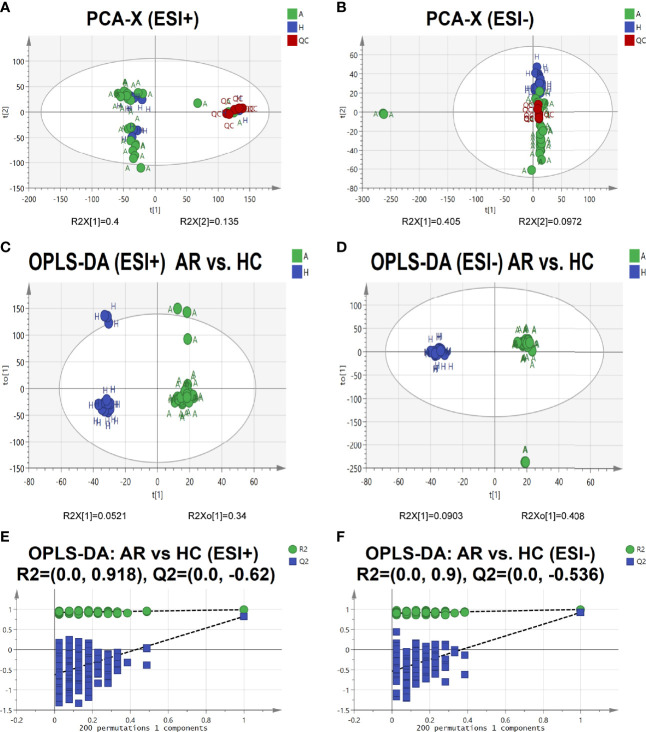
Serum metabolomic profile in different groups. **(A, B)** PCA score plots of serum metabolic profiling of allergic rhinitis and healthy control groups. **(C, D)** OPLS-DA score plots of serum metabolic profiling of AR and HC groups. **(E, F)** The permutations plots of the OPLS-DA models.

To further identify the pathogenesis of allergic rhinitis, non-targeted metabolomics was performed using UPLC-QTOF-MS/MS. Principal component analysis (PCA) is an unsupervised pattern recognition method that could be applied to select distinct variables in the search of possible biomarkers. PCA score 2D plots of the serum metabolomics in ESI+ and ESI- modes were displayed in **Figures 5A, B**. QC samples were tightly clustered, which further indicated the good stability of the metabolomics system. Moreover, a clear separation of the allergic rhinitis group (AR) and healthy control group (HC) could be observed, indicating that these two groups were differential.

Orthogonal Projections to Latent Structures Discriminant Analysis (OPLS-DA), a supervised method of pattern recognition, enables visualization and depiction of the general metabolic variation between the AR and HC groups. As shown in **Figures 5C, D**, each sample was represented as one spot in score plots and the AR and HC groups were separated in ESI+ and ESI- modes. Permutation tests (n=200) were used to validate the OPLS-DA model. From the permutation plots, all blue Q2-values to the left were lower than the original points to the right, indicating the validity of the original models (see **Figures 5E, F**).

#### 3.3.2 Identification of the Differential Metabolites in Serum

**Figure 6:**
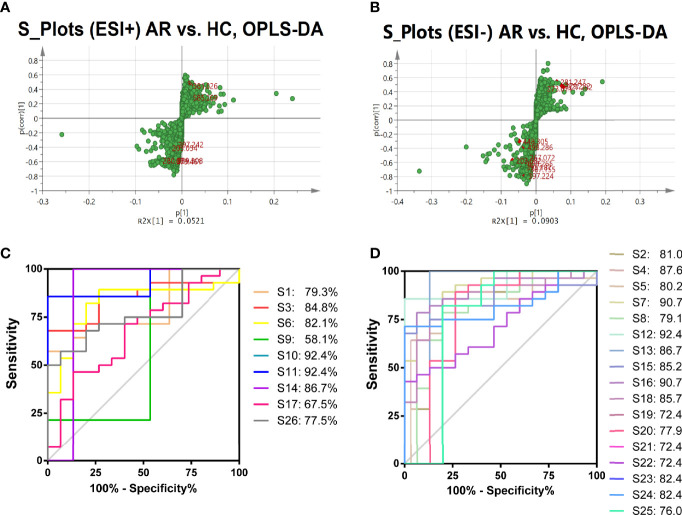
Identification of the Differential Metabolites in Serum. A-B, OPLS-DA S-plots of metabolomic analysis based on serum samples in ESI+ mode **(A)** and in ESI- mode **(B)**. The predictive ROC curves generated using 26 candidate biomarkers contributing to AR progress. **(C)** C_AR_>C_HC_; **(D)** C_AR_<C_HC_.

S-plots were created to identify differential metabolites (**Figures 6A, B**). Different spots in the S-plots represented different variables, and the farther away they were from the origin, the more significantly the spots contributed to the difference between the disease group and healthy group. Potentially differential metabolites were selected based on the contribution of Variable Importance for the Projection (VIP) extracted from the OPLS-DA models above. A total of 26 robust endogenous metabolites in serum were identified as potential biomarkers (marked in red in S-plots) based on VIP>1.0, p<0.05 standard screening and mass spectrometry comparison. After comparison with the referenced spectra from HMDB or METLIN databases of every metabolite, where the detailed information of fragments used to identify was shown in **Table 2**. Predicted ROC curves were generated using 26 candidate biomarkers in ESI+ and ESI- modes, suggesting that these metabolites are potential diagnostic markers for allergic rhinitis (**Figures 6C, D**).

**Table 2:** Distinct metabolites identified in serum samples.

No.	Compound Name	Formula	Rt/min	Mass/Da	KEGG ID	VIP	ESI	Error/ppm	Pathways	Content Level
S1	Deoxyuridine	C9H12N2O5	0.74	273.071	C00526	1.12	–	7	Pyrimidine metabolism	C_HC_<C_AR_
S2	Inosine	C10H12N4O5	0.8	267.072	C00294	1.59	–	6	Purine metabolism	C_AR_<C_HC_
S3	Oleic acid	C18H34O2	26.43	281.247	C00712	3.16	–	6	Biosynthesis of unsaturated fatty acids	C_HC_<C_AR_
S4	Coproporphyrin III	C36H38N4O8	7.7	653.265	C05770	1.89	–	5	Porphyrin and chlorophyll metabolism	C_AR_<C_HC_
S5	Chenodeoxycholic acid glycine conjugate	C26H43NO5	17.15	448.305	C05466	2.62	–	4	Primary bile acid biosynthesis	C_AR_<C_HC_
S6	Linoleic acid	C18H32O2	24.97	279.232	C01595	3.90	–	3	Linoleic acid metabolism; Biosynthesis of unsaturated fatty acids	C_HC_<C_AR_
S7	L-Tryptophan	C11H12N2O2	2.12	203.082	C00078	3.64	–	3	Tryptophan metabolism; Aminoacyl-tRNA biosynthesis	C_AR_<C_HC_
S8	Taurochenodesoxycholic acid	C26H45NO6S	15.55	498.286	C05465	1.84	–	7	Primary bile acid biosynthesis	C_AR_<C_HC_
S9	Prostaglandin E2	C20H32O5	22.55	397.224	C00584	1.74	–	2	Arachidonic acid metabolism	C_HC_<C_AR_
S10	Prostaglandin H2	C20H32O5	22.55	397.224	C00427	1.74	–	2	Arachidonic acid metabolism	C_HC_<C_AR_
S11	Prostaglandin D2	C20H32O5	22.55	397.224	C00696	1.74	–	2	Arachidonic acid metabolism	C_HC_<C_AR_
S12	Thromboxane A2	C20H32O5	22.55	397.224	C02198	1.74	–	2	Arachidonic acid metabolism	C_AR_<C_HC_
S13	20-Hydroxy-leukotriene B4	C20H32O5	22.55	397.224	C04853	1.74	–	2	Arachidonic acid metabolism	C_AR_<C_HC_
S14	Docosahexaenoic acid	C22H32O2	24.38	327.232	C06429	4.24	–	3	Biosynthesis of unsaturated fatty acids	C_HC_<C_AR_
S15	Pregnenolone sulfate	C21H32O5S	15.77	395.187	C18044	2.07	–	7	Steroid hormone biosynthesis	C_AR_<C_HC_
S16	Dehydroepiandrosterone sulfate	C19H28O5S	14.82	367.155	C04555	1.47	–	9	Steroid hormone biosynthesis	C_AR_<C_HC_
S17	Bilirubin	C33H36N4O6	11.92	585.269	C00486	1.12	+	3	Porphyrin and chlorophyll metabolism	C_HC_<C_AR_
S18	Glycerophosphocholine	C8H20NO6P	0.61	280.094	C00670	3.64	+	7	Ether lipid metabolism; Glycerophospholipid metabolism	C_AR_<C_HC_
S19	D-Glucose	C6H12O6	0.66	203.054	C00221	2.11	+	7	Glycolysis/Gluconeogenesis	C_AR_<C_HC_
S20	Presqualene diphosphate	C30H52O7P2	5.49	609.308	C03428	1.35	+	0	Steroid biosynthesis	C_AR_<C_HC_
S21	Paraxanthine	C7H8N4O2	0.66	203.054	C13747	2.11	+	0	Caffeine metabolism	C_AR_<C_HC_
S22	Theobromine	C7H8N4O2	0.66	203.054	C07480	2.11	+	0	Caffeine metabolism	C_AR_<C_HC_
S23	9,10-Epoxyoctadecenoic acid	C18H32O3	21.19	297.242	C14825	1.16	+	1	Linoleic acid metabolism	C_AR_<C_HC_
S24	12,13-EpOME	C18H32O3	21.19	297.242	C14826	1.16	+	1	Linoleic acid metabolism	C_AR_<C_HC_
S25	PA(P-16:0/18:2(9Z,12Z))	C37H69O7P	15.2	679.461	C15647	1.50	+	9	Ether lipid metabolism	C_AR_<C_HC_
S26	6-Thioxanthine 5’-monophosphate	C10H13N4O8PS	0.62	381.026	C16618	1.11	+	1	Drug metabolism - other enzymes	C_HC_<C_AR_

RT, Rentention Time; Mass, Measured mass.

#### 3.3.3 Identification of the Differential Metabolic Pathways in Serum

**Figure 7:**
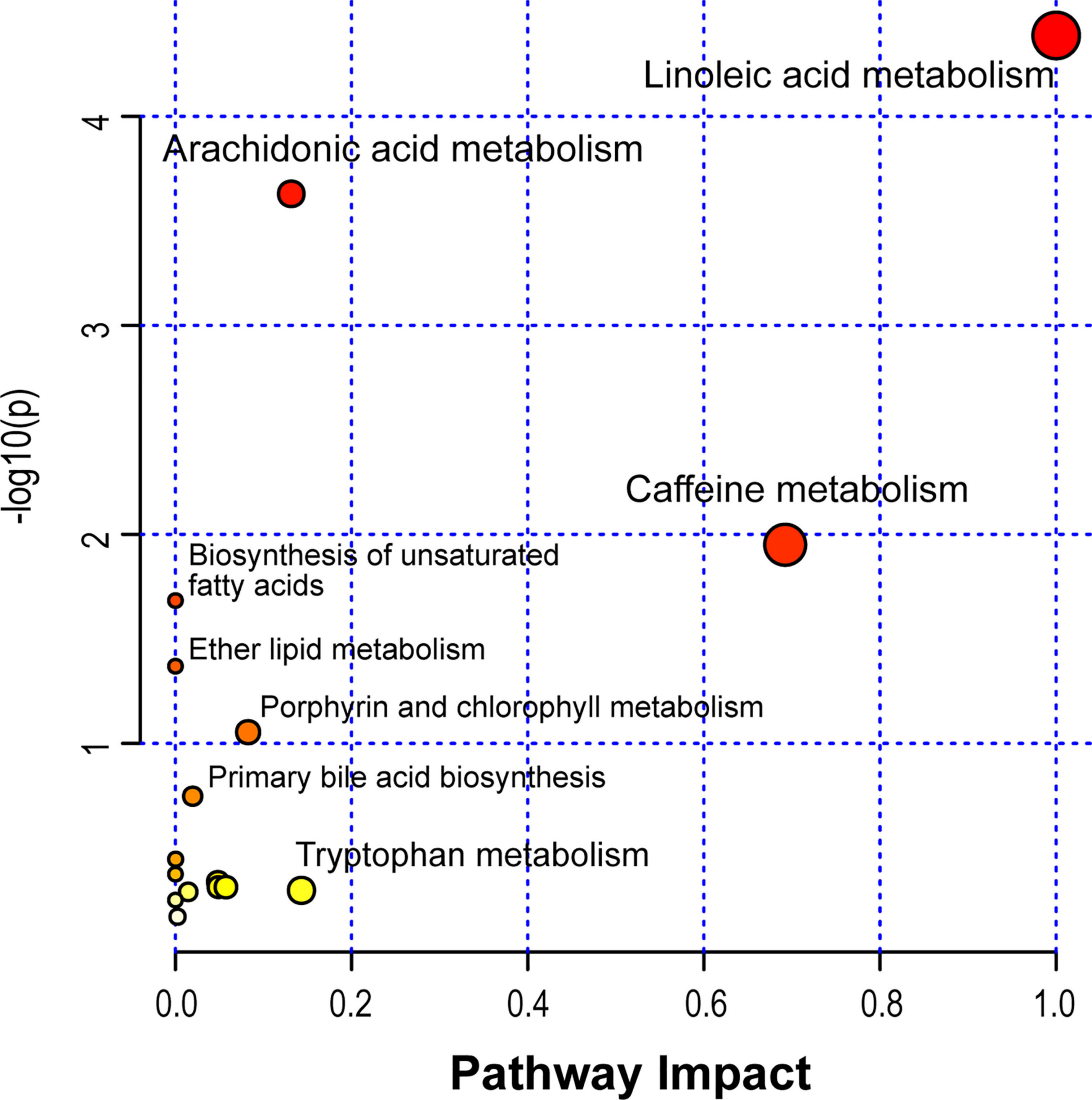
Disturbed metabolic pathways in serum samples were performed by MetaboAnalyst 4.0.

Based on the identified metabolites, 16 disturbed metabolic pathways that may be associated with the occurrence of AR were identified using MetaboAnalyst 4.0 and the results are shown in **Figure 7**. The impact value represents the importance of the metabolic pathways, the -log10(P) value represents the difference in the metabolic pathway and the size of the circle is positively correlated with the two parameters mentioned above. The identified metabolic pathways are summarized in **Table 3**. A total of three significantly altered pathways were observed in the serum samples, specifically, two altered metabolic pathways (linoleic acid metabolism and arachidonic acid metabolism) showed extreme significance (impact > 0.1, p<0.01), and the remaining one pathway (caffeine metabolism) showed potential significance (impact > 0.1, p < 0.05). Whereas tryptophan metabolism was potentially correlated with the occurrence of AR (impact > 0.1, p > 0.05).

**Table 3:** The results from metabolic pathways of distinct metabolites.

Pathway Name	Match Status	p	-log_10_(P)	FDR	Impact
Linoleic acid metabolism	3/5	4.10E-05	4.3868	0.0034476	1
Arachidonic acid metabolism	5/36	0.0002349	3.6291	0.0098659	0.13147
Caffeine metabolism	2/10	0.011214	1.9502	0.31399	0.69231
Biosynthesis of unsaturated fatty acids	3/36	0.020733	1.6833	0.4354	0
Ether lipid metabolism	2/20	0.042722	1.3693	0.71774	0
Porphyrin and chlorophyll metabolism	2/30	0.088342	1.0538	1	0.08243
Primary bile acid biosynthesis	2/46	0.17897	0.74722	1	0.01954
Glycolysis/Gluconeogenesis	1/26	0.35818	0.4459	1	0.00021
Steroid hormone biosynthesis	2/85	0.42225	0.37443	1	0
Glycerophospholipid metabolism	1/36	0.45992	0.33732	1	0.04814
Drug metabolism - other enzymes	1/39	0.48729	0.31222	1	0.04891
Pyrimidine metabolism	1/39	0.48729	0.31222	1	0.05729
Tryptophan metabolism	1/41	0.50478	0.29689	1	0.14305
Steroid biosynthesis	1/42	0.51332	0.28961	1	0.01444
Aminoacyl-tRNA biosynthesis	1/48	0.56162	0.25056	1	0
Purine metabolism	1/65	0.67474	0.17086	1	0.00234

### 3.4 Microbiome and Metabolome Association Analysis in AR

**Figure 8:**
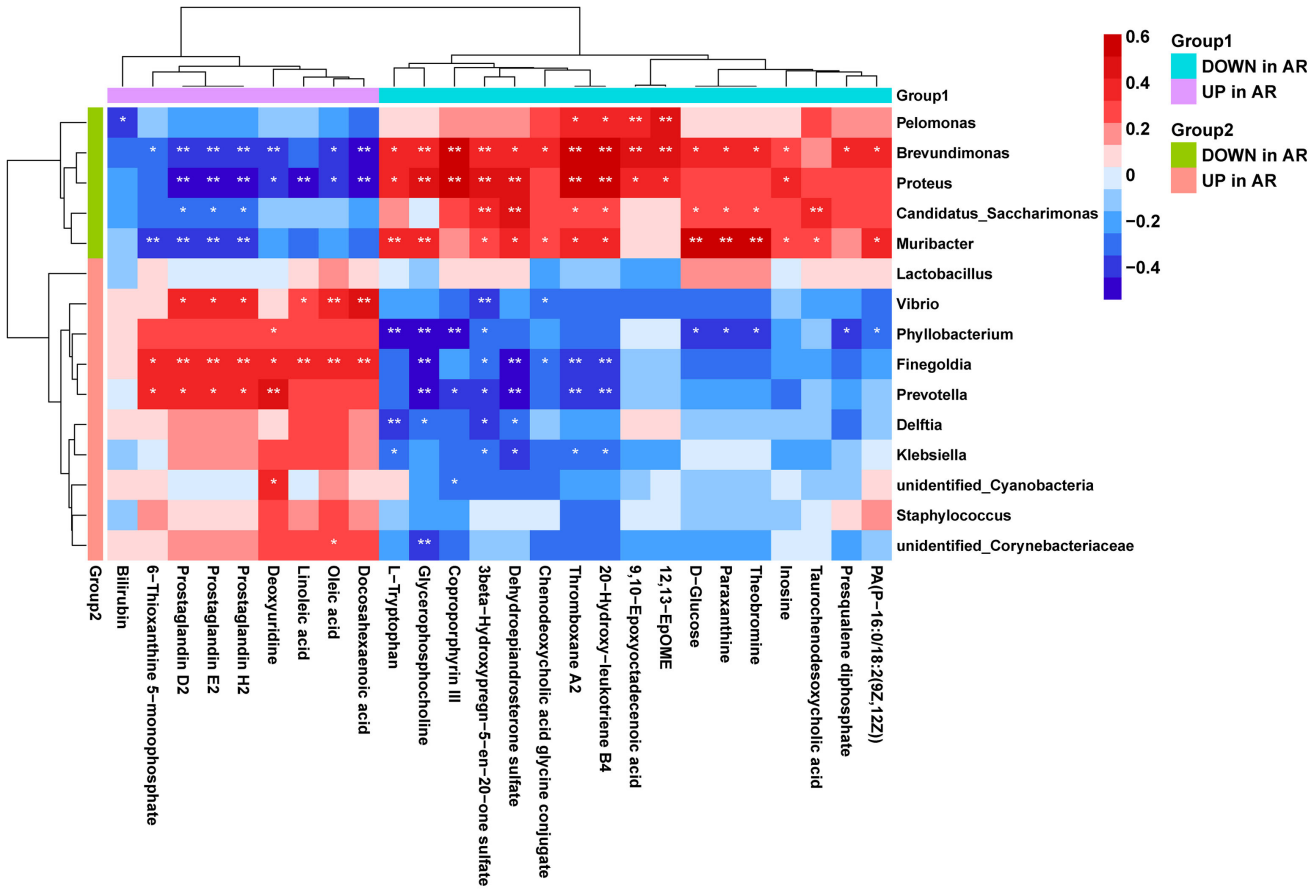
Correlation analysis of the microbiome and metabolome. Spearman’s correlation analysis was conducted using potential microbiome biomarkers of AR and potential metabolite biomarkers. Student’s t-test, *p < 0.05, **p < 0.01.

Using correlation analysis between microbiome and metabolome, we calculated the Spearman’s correlation between the 15 differential genera and the 26 differential metabolites (**Figure 8** **)**. We found that the AR-enriched genera correlated positively with AR-enriched metabolites but negatively with the HC-enriched metabolites. Consistently, the HC-enriched genera correlated positively with HC-enriched metabolites but negatively with AR-enriched metabolites, implying highly consistent metabolic interactions between the respiratory microbiota and the host. *Brevundimonas*, *Proteus*, *Muribacter*, *Finegoldia* and *Prevotella* had the most significant relationships with metabolites. In contrast, *Lactobacillus* and *Staphylococcus* were barely associated with differential metabolites.

## 4 Discussion

In this study, we first collected nasal inferior turbinate swabs from AR patients and HC volunteers, and combined with high-throughput sequencing of 16S rRNA reported no significant differences in diversity, abundance and homogeneity between the disease and control groups, but changes in microbiota structure, which revealed significantly higher levels of 1 phylum and 7 genera and significantly lower levels of 1 genus in the AR group compared to the HC group. Based on the characteristics of OTU we constructed a random forest prediction model with 10 genera and performed 10-fold cross-validation of the model, indicating that the airway microbiota has the potential to diagnose patients with allergic rhinitis and may be used as a candidate biomarker. We then used metabolomics to analyze serum from AR patients and HC volunteers and found that the systematic metabolic profile of AR patients was altered, identifying a total of 26 different metabolites and 16 perturbed metabolic pathways. The multi-omics study revealed that the upper respiratory microbiota maintains a highly consistent role with host metabolism.

Microbiota plays an essential role in regulating the immune response associated with atopic diseases. We noted that the inferior turbinate microbiota of AR patients in the acute exacerbation phase consists primarily of members of the phyla *Proteobacteria*, *Firmicutes*, and *Bacteroidetes*, which are similar to the that of HC (29, 30), but differs in that the phyla *Actinobacteria* is significantly increased in AR. And on a lower taxonomic level, the genera *Klebsiella*, *Prevotella*, and *Staphylococcus* were significantly increased in AR, while in HC, the most prevalent genera were *Moraxella*, *Haemophilus*, *Streptococcus*, and *Flavobacterium* (31, 32), differing probably due to dysbiosis of microbial ecology caused by the interaction between inflammatory state and microorganisms. Under normal conditions, the microbiota of the nasopharynx are predominantly *Proteobacteria* (e.g., *Moraxella* spp. and *Haemophilus* spp.), *Firmicutes* (e.g., *Staphylococcus* and *Dolosigranulum* spp.), and *Actinobacteria* (e.g., *Corynebacterium* spp.) (33, 34). The increase of the phyla *Actinobacteria* and the genera *Klebsiella*, *Prevotella* and *Staphylococcus* in the upper respiratory tract of AR patients may be related to the enlargement of the inferior turbinates during the allergic state, increasing epithelial permeability, allowing the presence of abnormal fluid accumulation on the airway surface and affecting the balance of the commensal microbiota in the nasal mucosa. *Klebsiella* has two main channels of colonization in the population host: the upper respiratory tract and the intestine. Its colonization fights against the microbiota in these two locations and the defenses established by the immune system. It has been shown that in the upper respiratory tract, *Proteobacteria* enhances immunity through IL-17A, but *Klebsiella* overcomes these defenses and thus colonizes effectively by encapsulation (35). *Bacteroidetes* are sufficient to prevent the host-to-host spread of *Klebsiella* between hosts *via* IL-36 (35). *Staphylococcus* are common in patients with allergic rhinitis, especially *S. aureus*, with a colonization rate of 44% compared to 20% in healthy controls (36). And allergic *S. aureus* carriers had higher nasal symptom scores (37). Hyun et al. described a lower microbial biodiversity observed in individuals with high serum total IgE levels (high IgE group) compared to individuals with low total IgE levels (low IgE group), with a higher relative abundance of *Staphylococcus aureus* (38). *Staphylococcus aureus* induces IgE production and promotes allergic inflammation. High IgE levels cause *S. aureus* blooms, which activate mast cell degranulation and lead to inflammation. Therefore, controlling *S. aureus* and IgE levels may be an effective strategy to prevent IgE-related diseases, including AR. *Prevotella* is the second most abundant genus in the human oral cavity, and it is essentially the most abundant of the gut microbiota whenever it is present. the association between *Prevotella* and diet may account for its decline in westernized populations, whose diets are rich in fat and fiber (39). Chiu et al. suggested that airway microbial dysbiosis in response to HDM and its interaction with intestinal microbiota are associated with early allergic respiratory disease in children (40). Thus, the dietary modulation of gut microbiota was suggested to be involved in allergy processes (41, 42) and to influence the microbiota alterations in the airway and gut. Not only adults, but also children with chronic rhinitis have been shown to have significant differences in the relative abundance of specific microbiota compared to children with healthy conditions (34, 43). These results support our findings that the IgE mediated inflammatory response characteristic of AR may influence the airway microbiota. The potential for variability shown by differentially abundant taxa provides a starting point for future studies with the potential to improve patient outcomes. However, the results are inconsistent as to whether there are differences in the diversity of airway microbiota between AR and HC (38, 44), which may be due to the different regional environments, different types of allergens and different amounts of allergens in the study population.

Metabolomics is a branch of omics techniques that systematically analyzes the concentration of all low molecular mass metabolites in the organism (45). Metabolites provide the opportunity to establish powerful exploratory tools for monitoring disease states and help to explain the pathophysiological mechanisms of disease (46). Metabolomic profiling from serum or urine samples has been widely applied to many diseases to identify markers for early disease detection and treatment outcome prediction (22, 23). Previously, metabolomic profiles of urine have been demonstrated to distinguish between healthy children and asthmatics (47), between patients with unstable asthma in the emergency room and patients with stable asthma in the clinic, and between adults with asthma and patients with chronic obstructive pulmonary disease (48). In the present study, we have reported the differential metabolic profile between AR patients and healthy subjects. All differential metabolites in serum were identified with UPLC-MS/MS techniques. Multivariate analysis was performed to clarify the difference between the two groups. We developed robust and reliable OPLS-DA models characterizing 26 different metabolites and 16 perturbed metabolic pathways. Linoleic acid metabolism, arachidonic acid metabolism and caffeine metabolism, the top 3 significantly altered metabolic pathways, have been determined. Notably, arachidonic acid (AA) metabolism network produces crucial inflammatory mediators that are notably considered to be hallmarks of diverse inflammation-related diseases, including allergic asthma (49). We found significantly altered metabolites related to arachidonic acid metabolism in the serum of AR patients, suggesting that the formation and development of AR may be associated with abnormal metabolism of AA. In the AA/Cyclooxygenase (COX) pathway, the hematopoietic PGD synthase (hPGDS) catalyzes the isomerization of PGH2 to PGD2, which makes it an interesting target for the treatment of allergic inflammation (50). In the present study, Prostaglandin (PG) D2 levels were significantly higher in AR patients than in healthy volunteers, suggesting that PGD2 signaling may be a promising biomarker, as IgE binding to mast cells triggers the release of PGD2, which activates eosinophils and basophils. Zhou et al. studied serum samples from pollinosis patients using NMR-based metabolic patients and found that pollinosis could alter the metabolic profile of energy, amino acid and lipid in patients, which may be diagnostic and/or prognostic markers in patients with hay fever (51). Adamko et al. provided conceptual evidence that metabolomic analysis of excreted urine metabolites could distinguish severity in patients with AR (52). A recent serum metabolomics study showed that at least nine metabolites (13(S)-HPODE, bilirubin, leukotriene D4, hypoxanthine, L-steroidal bilirubin, N-succinyl-L-diaminoheptanedioic acid, chlorophyllb, 15-hydroxyeicosatetraenoic acid, and uric acid) were significantly altered in the serum of patients with AR (53). Another study identified serum biomarkers that can reliably and correctly predict the efficacy of sublingual immunotherapy in patients with AR (54). These studies may contribute to a better understanding of the underlying pathogenesis and provide metabolic evidence for in-depth studies of AR.

Integrative analysis of microbiomes and non-target metabolomes of diseased individuals preliminary revealed the relationship between differential microorganisms and differential metabolites and indicated two major lipid metabolic pathways, linoleic acid and arachidonic acid metabolism. Our multi-omics study demonstrated the correlation between differential bacterial genera and metabolites. While the causes of these differentially expressed metabolites may or may not result from the altered microbiota structure, the nutritional homeostasis may also be at play at the host-microbiome interface (55). We hypothesize that the altered metabolites and immune environment during acute exacerbations of allergic rhinitis promotes increased colonization of the respiratory tract by harmful bacteria such as *Klebsiella* and *Staphylococcus*, and that host-microbiota interactions affect the host immune system, influencing metabolic pathways such as linoleic acid metabolism and arachidonic acid metabolism and promoting allergic responses. There is emerging evidence that bacterial metabolites, toxins and structural components from pathogenic and opportunistic bacteria could stimulate detrimental immune responses that contribute to the pathogenesis of respiratory disease (56). Our research provides strong evidence for an in-depth study of the mechanisms of AR, but there are still significant limitations. The sample size of this study is small and the future studies with increased sample size are necessary to further elucidate the roles of the identified factors and lipid and metabolic pathways in allergic rhinitis exacerbation. Allergic rhinitis can be classified into multiple subtypes, such as seasonal/perennial, intermittent/persistent (57), monosensitized/polysensitized (58) and mild/moderate/severe (59). In the future, the search for specific, sensitive and reliable biomarkers for different subtypes of allergic rhinitis patients will open new avenues for more precise disease classification and individualized targeted therapy.

## 5 Conclusion

In conclusion, there are differential microorganisms with different relative abundance and structural composition in the upper airways of AR patients compared to those of HC volunteers. Understanding the role of the airway microbiota may help to modulate the therapeutic strategy for AR. The current metabolomic study showed the presence of 26 identifiable differential metabolites and 16 perturbed metabolic pathways between AR patients and HCs, which involve immune regulation. The identified metabolites contributed to the understanding of the pathophysiology of allergic rhinitis, and further targeted metabolomics are needed to improve therapeutic strategies.

## Data Availability Statement

The datasets presented in this study can be found in online repositories. The names of the repository/repositories and accession number(s) can be found below: https://www.ncbi.nlm.nih.gov/, PRJNA760816.

## Ethics Statement

The studies involving human participants were reviewed and approved by China - Japan Union Hospital of Jilin University. The patients/participants provided their written informed consent to participate in this study.

## Author Contributions

FW designed and supervised the study. YY reviewed the literature and wrote the manuscript. CW, GW, XG, and SJ performed bioinformatics analysis, statistical analysis, and completed graphing. XZ and XW recruited patients and registered clinical information. AH and MQ revised the manuscript. All authors contributed to the article and approved the submitted version.

## Funding

This study was funded by the Jilin Provincial Key Laboratory of Precision Infectious Diseases (Grant No. 20200601011JC), Key Laboratory of Health and Family Planning Commission of Jilin Province (Grant No. 3D5200117426), NMPA Key Laboratory of Humanized Animal Models for Evaluation of Vaccines and Cell Therapy Products and Graduate Innovation Fund of Jilin University (Grant No. 101832018C060).

## Conflict of Interest

The authors declare that the research was conducted in the absence of any commercial or financial relationships that could be construed as a potential conflict of interest.

## Publisher’s Note

All claims expressed in this article are solely those of the authors and do not necessarily represent those of their affiliated organizations, or those of the publisher, the editors and the reviewers. Any product that may be evaluated in this article, or claim that may be made by its manufacturer, is not guaranteed or endorsed by the publisher.
